# Chemotherapy Drug Induced Discoordination of Mitochondrial Life Cycle Detected by Cardiolipin Fluctuation

**DOI:** 10.1371/journal.pone.0162457

**Published:** 2016-09-14

**Authors:** Yu-Jen Chao, Jui-Fen Chan, Yuan-Hao Howard Hsu

**Affiliations:** 1 Department of Chemistry, Tunghai University, Taichung, Taiwan; 2 Life Science Research Center, Tunghai University, Taichung, Taiwan; Universidad Pablo de Olavide, SPAIN

## Abstract

Chemotherapy drugs have been prescribed for the systemic treatment of cancer. We selected three chemotherapy drugs, including methotrexate, mitomycine C and vincristine to inhibit the proliferation of HT1080 human fibrosarcoma cells in S, G2 and M phases of the cell cycle respectively. These chemotherapy drugs showed significant toxicity and growth inhibition to the cancer cells measured by MTT assay. After treated with a 50% inhibitory dosage for 48 hours, these cancer cells showed significant accumulation of cardiolipin (CL), which was a reverse trend of the nutritional deficiency induced arrest at G1 phase. The quantity of each CL species was further semi-quantitated by HPLC-ion trap mass spectrometer. Methotraxate treatment caused unique increases of acyl chain length on CL, which were the opposite of the serum starvation, mitomycine C and vincristine treatments. Although mitomycine C and vincristine have different mechanisms to induce cell cycle arrest, these two drugs displayed similar effects on decreasing chain length of CL. Continuation of CL synthesis during cell cycle arrest indicated the chemotherapy drugs resulting in the discoordination of the mitochondrial life cycle from the cell cycle and thus caused the accumulation of CL. These finding reveals that the pre-remodeling nascent CL accumulates during the methotraxate induced arrest; however, the post-remodeling mature CL accumulates during the mitomycine C and vincristine induced arrest after the synthesis phase.

## Introduction

Chemotherapy drugs are often designed to interfere with cell growth signals, inhibit enzymatic activities or compete with the required materials for replication in various phases of the cell cycle to achieve cell cycle arrest and prevent cancer progression [1–3]. When cells sustain DNA damage, nutritional deficiency or oxidative stress, the cell cycle is arrested at the check points. The cell cycle is highly regulated by Cyclin-CDK complex [4–6]. Inhibition of the CDK(s) by CDK interacting protein/kinase inhibitory protein family (cip/kip) [7] and the inhibitor of kinase 4/alternative reading frame family (INK4a/ARF) [8,9] would trigger cell cycle arrest. When the cells encounter the irreversible DNA damage or the unrepairable cell damage, the cells would escape or skip the arresting state, leading to apoptosis or necrosis eventually [10,11]. The FDA-approved chemotherapeutic drugs, methotrexate, mitomycine C and vincristine, have been clinically applied to treat the unregulated cancer cells through different cellular targets and mechanisms to interfere with the cell cycle. The methotrexate is an analog of dihydrofolic acid, which arrests the cells in S phase [12–14]. Mitomycine C arrests cells in G2/M phase through binding to guanine in minor grove in DNA [15,16]. Vincristine prevents the polymerization of microtubule to arrest cell in M phase [17–19].

Synthesis and remodeling of phospholipid along the cell cycle regulate the integrity and functions of the organelle membranes [20]. Phospholipids turnover for maturation is active in G1 phase [21–23]. The synthesis of phospholipids is effective in S phase and finished for mitosis in G2/M phase [24]. Normal growing cells usually have a good coordination between the cell cycle and the mitochondrial life cycle to regulate cell growth and mitochondrial dynamics [25,26]. The unregulated cancer cells shift ATP demand from oxidative phosphorylation to glycolysis, but high mitochondrial activity is still required to produce intermediates to support cell proliferation, which induces variations of the mitochondrial property [27]. Recent studies have also indicated mitochondria to be a good chemotherapy target [28,29]. Inhibition of cell growth during chemotherapy treatment inevitably disturbs the mitochondrial life cycle, although how the arrest of the cancer cells affecting the coordination of the two cycles and the subsequent phospholipid metabolism is still not clear.

Cardiolipin (CL) is a mitochondrial phospholipid synthesized in mitochondria and involved in the maintenance of the mitochondrial architecture and the stabilization of the ETC complexes for ATP production [20,26,30–37]. Maturation of the nascent CL includes multiple steps of synthesis and remodeling in a mitochondrial life cycle [38]. The acquisition of the CL symmetry from remodeling of nascent CL by tafazzin is critical for the function of mitochondria [39]. It has been found that dysregulation of the synthesis and remodeling of CL resulted in the loss of the symmetry in rat brain tumor [40]. Loss of symmetry by oxidation and hydrolysis of CL has further been discovered in cancer, mitophagy or further downstream apoptosis [41,42]. The CL species inside mitochondria are also dependent on the environmental conditions. The nutritional deficiency and the exogenous nutritional supplementation dynamically changed the species content of the CL [43,44]. The cardiolipin species shortened their chain length during cell cycle arrest induced by the nutritional deficiency [43]. Recovery of the cell growth from serum starvation stimulated the synthesis of CL to regain the longer acyl chains. The dynamic of CL contents were also found in incorporation of the supplementary EPA and DHA into CL to change CL species in mitochondria in H9c2 cells [44].

Because CL is a critical phospholipid related to ATP production for cancer cell progression, we are interested in understanding how the CL changes dynamically after the cell cycle arrested in S, G1 and G2/M phases by chemotherapeutic drugs. We have previously identified 40 CL species in HT1080 and H9c2 cells by mass spectrometry [43,44]. Here we applied this technique to extend our understanding on the CL dynamics in the cell cycle upon treatments of chemotherapy drugs.

## Materials and Methods

### Materials

Dulbecco's modified eagle medium (DMEM), fetal bovine serum (FBS), penicillin/streptomycin and 1M HEPES for cell culture were purchased from Gibco, USA. Tetramyristoyl cardiolipin standard CL(14:0)_4_ and tetralinoleoyl cardiolipin CL(18:2)_4_ extracted from bovine heart were purchased from Avanti Polar Lipids, USA. Methotrexate and mitomycin C were bought from Merck Co. (Darmstadt, Germany). Vincristine sulfate was acquired from Sigma Chemical Co. (St. Louis, MO, USA).

### Cell Starvation

HT-1080 human fibrosarcoma cells (American Type Culture Collection) were cultured in Dulbecco's modified eagle medium (DMEM) with 10% fetal bovine serum, 0.5% penicillin-streptomycin and 25 mM HEPES in 5% CO_2_ at 37°C. The cells were grown to 50% confluent in 6-cm culture dishes for starvation treatment, removed the medium and washed with PBS. The cells were then cultured in serum-free medium for 48 hr in triplicates. During the time course of serum starvation, the cells were harvested at 0, 12, 24, 36 and 48 hr.

### MTT assay

HT-1080 cells were cultured in DMEM with 10% fetal bovine serum in 5% CO_2_ at 37°C. The 5.0 X 10^4^ cells were transferred to a 24-well culture plate and let cells to attach to the dish surface to reach 25% confluence. The medium was replaced with the DMEM containing 25, 50, 100, 150 and 200 nM methotrexate, 1, 2, 4, 8 and 16 μM mitomycin C or 0.1, 0.2, 0.3, 0.4 and 0.5 nM vincristine. The drug treated cells were cultured for another 48 hours, washed with PBS, and added medium containing 0.5 mg/ml MTT(3-(4,5-dimethyldiazol-2-yl)-2,5 diphenyl tetrazolium bromide). After 4 hours of incubation at 37°C, MTT turns purple formazan. The medium was replaced with 200 μl of DMSO for 5 min. The absorbance of the dissolved cells in DMSO was measured at the wavelength 550 nm.

### Bradford assay

The harvested cells were washed with PBS, added 10 μl of 50% RIPA buffer (Thermo) and placed on ice for 5 min. After sonication for 30 sec, 490 μl of DDW was added to sample. 10 μl of the prepared sample was mixed with 190 μl of 1X solution of Bradford protein-binding assay (Bio-Rad) in 96-well plate. The absorbance at wavelength 595 nm of the plate was measured by SpectraMax M Series Multi-Mode Microplate Readers (Molecular Devices). The standard curve was based on BSA. The Bradford assay was used to quantify the total protein of the harvested cells. We calculated the average amount of the total protein per cell in the seeded 3 X 10^5^ cell in 6-cm culture dish. By quantifying the amount of protein per cell, we could estimate the cell number by Bradford assay.

### Chemotherapy Drug Treatments

HT-1080 cells were cultured in DMEM with 10% fetal bovine serum, 0.5% penicillin-streptomycin and 25 mM HEPES in 5% CO_2_ at 37°C. The 3.0 X 10^5^ cells were transferred to a 6-cm culture dish and let cells to attach to the dish surface to reach 25% confluence. The medium was replaced with the DMEM containing 50 nM methotrexate, 2 μM mitomycin C or 0.25 nM vincristine. The concentration of each chemotherapy drug was determined by 50% inhibition of cell growth after 48 hours of treatment in the MTT assay. After 0, 12, 24, 36 and 48 hours of incubation, the cells were harvested, washed with PBS and stored in -20°C for lipid extraction.

### Lipid Extraction

The total lipid in the collected cells was extracted according to the Bligh-Dyer’s method [45]. The internal standard, 125 ng tetramyristoyl cardiolipin CL(14:0)_4_, was added to the cell pallets in 2 ml methanol. After pulse sonication by 80W UP-80 ultrasonic processor (CT ChromTech, Taiwan) on ice, 1 mL of chloroform was added into samples and vortexed for 10 min. Then, 1 ml of chloroform and 1 ml of distilled deionized water were added to samples and further vortexed for 15 min. The lower phase in the glass test tube was collected after centrifugation at 2000 rpm for 5 min.

### Mass Spectrometry Analysis

The extracted total lipid was dried under nitrogen gas and immediately re-dissolved in 200 μl of acetonitrile/2-propanol/H_2_O (65:30:5). The samples were capped and stored in -20°C to prevent evaporation until the analysis by LC/MS Ion-Trap (Bruker Corporation). A total of 20 μl dissolved sample was injected through autosampler. HPLC mobile phases contained solution A: ACN:H_2_O (60:40), 10 mM ammonium formate, 0.1% formic acid and solution B: IPA:ACN (90:10), 10 mM ammonium formate, 0.1% formic acid [46]. Gradient was from 60% solution A to 100% solution B in 25 min and maintained 100% solution B until 45 min in an Acclaim RSLC 120 C18 2.1mm x 100 mm 2.2 μm column (Thermo) at a flow rate of 0.2 mL/min at 55°C. Data were further analyzed by Bruker DataAnalysis (ver.3.4). The extract ion current (XIC) of each cardiolipin species was quantitated by their relativity of XIC to internal standard. The total cardiolipin is the sum of all quantitated cardiolipin species. Standard deviations are calculated by the STDEV function in Microsoft Excel for the error bars of the histograms and t-tests are applied to all triplicated data (S1 Fig).

## Results

### Cell cycle arrest

**Fig 1:**
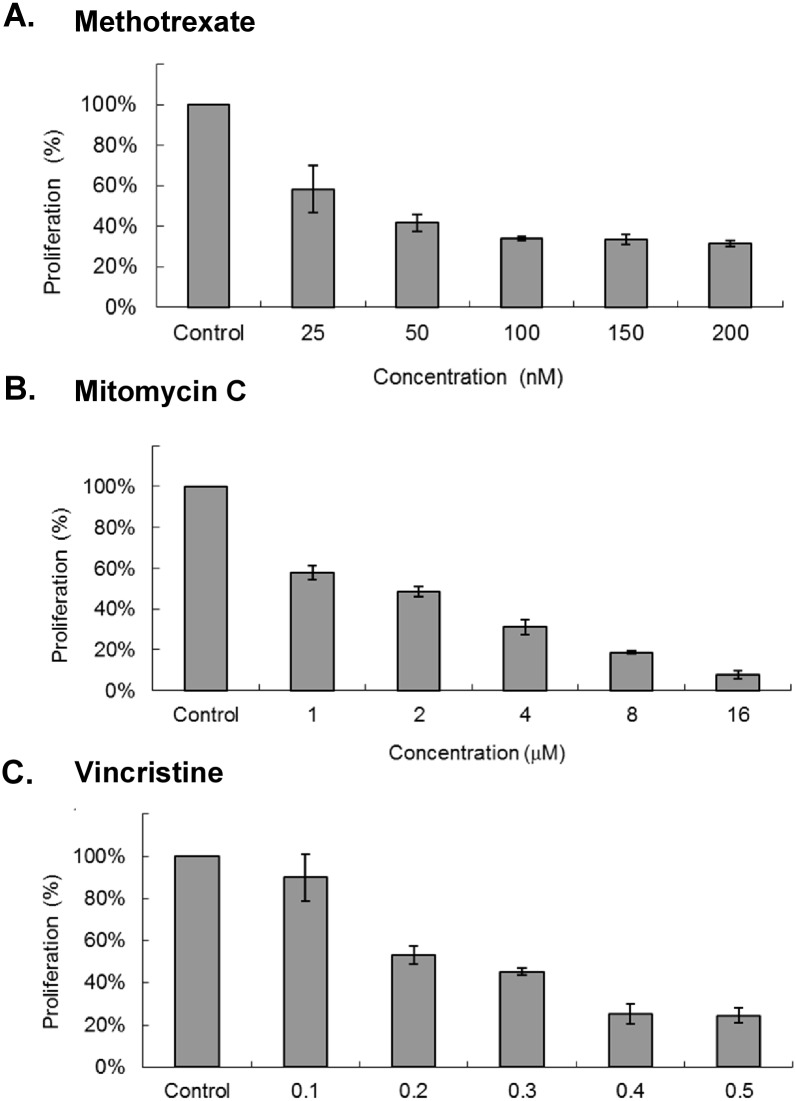
Cell viability after chemotherapeutic drug treatment. Cell viabilities were measured by MTT assays after treatment of (A) methotrexate, (B) mitomycin C and (C) vincristine on HT1080 cells for 48 hr. All experiments were in triplicates.

Inhibition of the cell cycle by chemotherapy drugs is arguable to perturb the coordinated mitochondrial life cycle. Therefore, we hypothesized that the discoordination of the two cycles would occur after chemotherapy drug induces cell cycle arrest. Under this hypothesis, the cell cycle can be inhibited in the drug treated cancer cells, but the mitochondrial life cycle is not arrested or responds accordingly. We could analyze the variation of the CL contents, including synthesis and remodeling in the arrested cells. To analyze the inhibition by chemotherapy drugs, methotrexate, mitomycin C and vincristine were selected to arrest the cells in S, G2 and M phases respectively. The CL contents in HT-1080 human fibrosarcoma cells have been shown to be responsive to nutritional deficiency induced cell cycle arrest [43]. Therefore, this cell line was chosen for further analysis of cell cycle arrest and CL remodeling in mitochondria. As we treated HT1080 cells with methotrexate, mitomycin C or vincristine, all drugs showed cellular toxicity measured by MTT assay (Fig 1). To observe CL remodeling at the arrested state, the concentrations of each drug with 50% inhibition of the mitochondrial activity tested in the MTT assays was applied to the cells for later experiments. At the concentration of 50 nM methotrexate, 2 μM mitomycin C and 0.25 nM vincristine, the drugs inhibited the cell growth, but did not cause cell death after two days of incubation. It is worth to note that the control was normal growing cells which have replicated twice and become 4 folds of the original seeded cells after 48 hours, but the drug treated cells reduced the growth rate and merely proliferated 1.2, 1.9 and 1.6 folds of the seeded cells.

### CL quantity change

**Fig 2:**
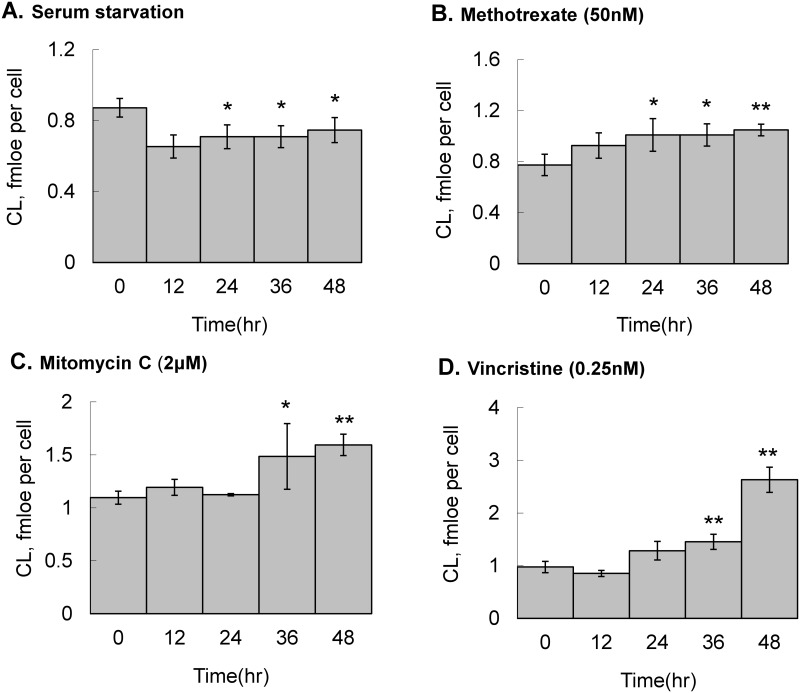
CL quantity changes in HT1080 after serum starvation and chemotherapy drug treatment. Cells were (A) serum deprived or treated with (B) 50 nM methotrexate, (C) 2 μM mitomycin C or (D) 0.25 nM vincristine and harvested at 0, 12, 24, 36 and 48 hr. After total lipid extraction, CL quantity in the 6-cm culture dish were determined by LC-MS in triplicates and analyzed by t-test (*p < 0.05, **p < 0.01, ***p < 0.001).

To observe the CL changes after chemotherapeutic drug treatment, HT1080 cells were serum deprived or stimulated with 50 nM methotrexate, 2 μM mitomycin C or 0.25 nM vincristine for 48 hr to reach cell cycle arrest. The cells were harvested every 12 hours and the contents of CL were analyzed by LC-MS (Fig 2). After 12-hr serum starvation, the harvested HT-1080 cells show a 20% decrease in total CL. The chemotherapy drugs, methotrexate, mitomycin C and vincristine, increase the quantity of CL significantly, which are the opposite effects of serum starvation. The accumulation of the CL quantity is significantly ascending through the 48 hr incubation, indicating accumulation of CL after treatment of chemotherapeutic drugs (Fig 2). Among these 3 drugs, methotrexate, mitomycin C and vincristine increased 35%, 45% and 169% respectively. Vincristine arrested cells after DNA synthesis phase to cause the most significant effects on CL quantity. Under the cell arresting state, the dramatic increase of the CL quantity means a continuation of phospholipid synthesis in mitochondria and is a good indication of the discoordination between the cell cycle and the mitochondrial life cycle.

### Serum starvation induced CL species change

The species of the CL previously identified by MS/MS were diverse in the HT1080 cancer cells [43]. The quantity of a particular species of CL was semi-quantitated by the relative XIC to standard CL (S2 Fig). The current HT-1080 cells contained majorly the CL groups of C68, C70 and C72, similar to that of our previous study. However, the relative intensity among three major CL groups changed between these two experiments. This effect is likely dependent on the health condition, the nutrition and the generation of the cells. Nevertheless, the cells used for experiments here displaying consistent CL pattern. For convenience, the sum of the carbon atoms on four acyl chains is used to indicate a particular group of CL with various double bonds. After 48 hours of serum starvation, the percentage of the major species C68, C70 and C72 of cardiolipin significantly shifted (S2 Fig), the same effects observed in our previous research [43]. Long chain C72 and C74 CL contents decreased and the short chain C66 and C68 CL increased. Serum deprivation shortened the chain length of CL.

### Methotrexate induced CL change

**Fig 3:**
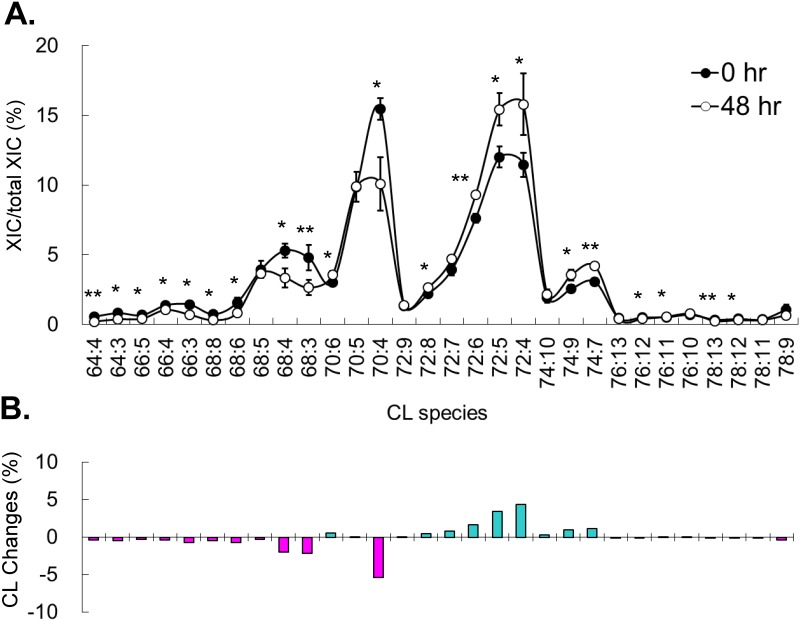
Methotrexate induced CL content changes. (A) Percentage of CL species in control and after 48-hr of methotrexate treatment. Experiments are in triplicates and analyzed by t-test (*p < 0.05, **p < 0.01, ***p < 0.001). (B) The changes of CL contents after 48-hr of methotrexate treatment. Total extracted ion current (XIC) is the total XIC of all detected CL.

Serum deprivation induces cell cycle arrest by nutritional deficiency, which uses a different mechanism from the chemotherapy drug for the arrest. Unlike serum deprivation, we observed accumulation of CL upon treatment of all 3 chemotherapy drugs. Because treatment of the chemotherapy drugs under serum deprivation at our experimental conditions caused cell death, CL changes in co-treatments were not examined in this study. Methotrexate is a dihydrofolic acid analog blocking dihydrofolic acid reductase and inhibits the synthesis of purines and pyrimidines, which are the necessary materials of DNA and RNA synthesis. Therefore, methotrexate has been shown to arrest cells in G1/S phase. To analyze the methotrexate induced CL changes, we collected the treated cells every 12 hours for 2 days. The changes ascended throughout the time course and the maximum differences at 48 hr are shown here (Fig 3). Methotrexate induced decreases of C66, C68 and C70 groups of short chain CL and increases of C72 and C74 groups of long chain CL. C72 group showed the highest increases of percentage, particularly CL72:4. The CL with lower number of the double bonds tended to have higher quantity increase. Compared to the serum starvation treatment, methotrexate induced cell cycle arrest triggered a reverse effect on both of the total quantity of CL and the remodeling effects on acyl chain length. Interestingly, the increases of the total CL and the chain length were similar to the effects on the serum replenish after starvation, indicating methotrexate treatment might cause the increase of mitochondria activity as the cell survival.

### Mitomycine C and vincristine effects on CL remodeling

**Fig 4:**
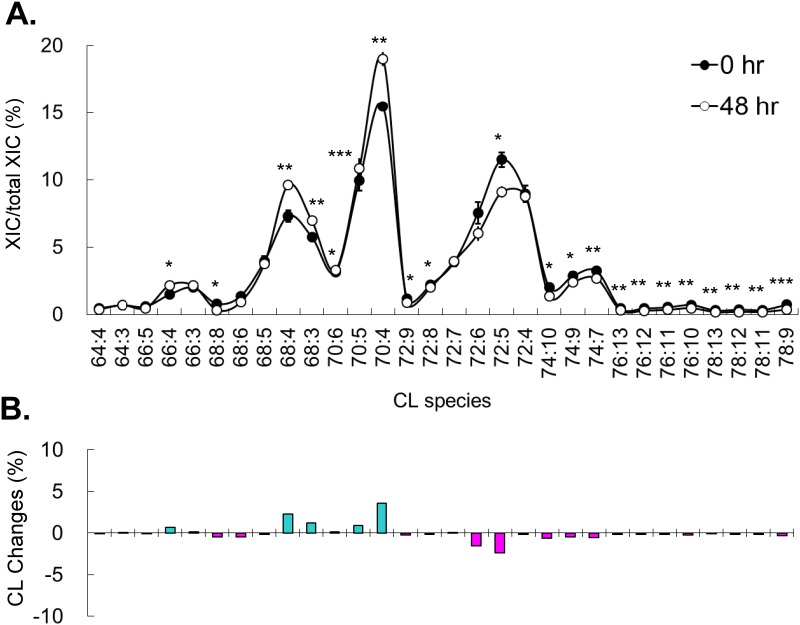
Mitomycine C induced CL content changes. (A) Percentage of CL species in control and after 48-hr of mitomycine C treatment. Experiments are in triplicates and analyzed by t-test (*p < 0.05, **p < 0.01, ***p < 0.001). (B) The changes of CL contents after 48-hr of mitomycine C treatment. Total extracted ion current (XIC) is the total XIC of all detected CL.

**Fig 5:**
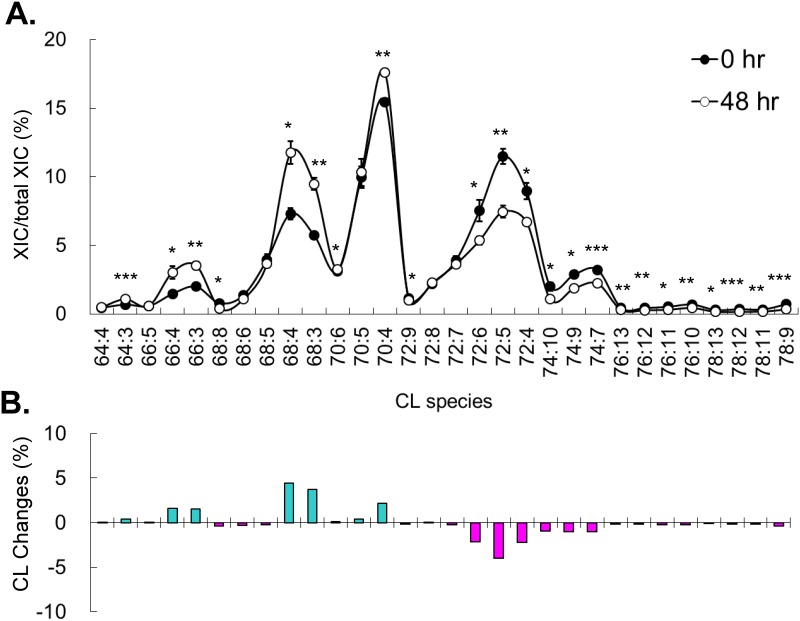
Vincristine induced CL content changes. (A) Percentage of CL species in control and after 48-hr of vincristine treatment. Experiments are in triplicates and analyzed by t-test (*p < 0.05, **p < 0.01, ***p < 0.001). (B) The changes of CL contents after 48-hr of vincristine treatment. Total extracted ion current (XIC) is the total XIC of all detected CL.

Methotrexate inhibits the cell cycle at the synthesis phase, but mitomycine C and vincristine inhibits after synthesis phase of the cell cycle. Mitomycine C and vincristine utilize two other individual mechanisms to inhibit the cell cycle; however, we observed two similar CL patterns after treatments of mitomycine C (Fig 4) and vincristine (Fig 5). Mitomicine C and vincristine induced increases of C66, C68 and C70 groups of short chain CL and decrease C72 and C74 groups of long chain CL. Mitomycine C and vincristine induced CL changes displayed a reverse trend of the methotrexate induced CL changes. Our results showed that mitomycine C and vincristine, which induce cell cycle arrest after synthesis phase caused a different CL pattern than methotrexate. This difference might be contributed by the CL remodeling immediately after the synthesis phase. Further comparison between the nutritional induced- and the drug induced-cell cycle arrest found G0 arrest is similar to the G2 and M phase arrest, except the C70 group, implying CL is not intensively remodeled between M and G1 phases.

### Comparisons of the cell arrest effects

**Fig 6:**
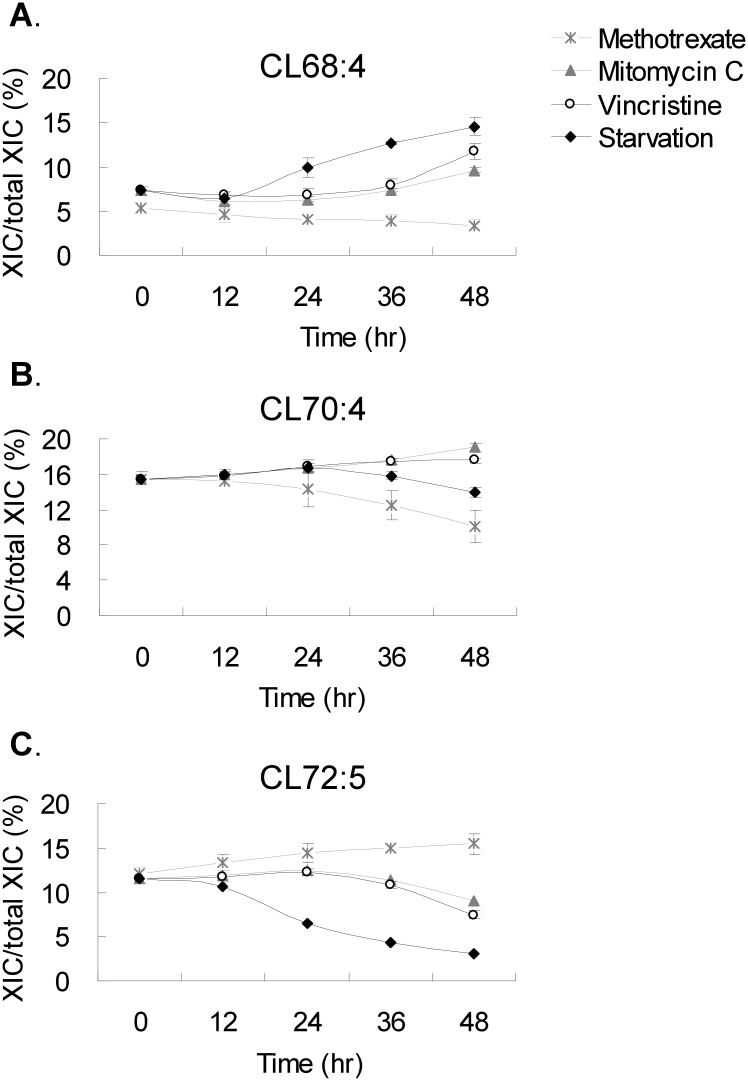
CL quantity changes of the main species upon chemotherapy drug treatment. The (A) C68:4, (B) C70:4 and (C) C72:5 are three most abundant CL species in the groups of CL containing 68, 70 and 72 carbons in the four acyl chains. The cells were treated with serum starvation, methotrexate, mitomycine C and vincristine for 0, 12, 24, 36 and 48 hr in triplicates.

We observed the difference of CL quantity between nutritional induced cell cycle arrest and the chemotherapy triggered cell cycle arrest. Each species of CL showed incremental change in the quantity over the 48-hour period of drug treatments. Three major species of CL representing C68, C70 and C72 groups displayed atypical trends of the changes over the 48-hour period (Fig 6). During the 48-hour drug treatment of inhibition, the significant changes were detected after 24 hours. From 24 hr to 48 hr, the trends continued and the differences elevated over time. The percentage of the short chain CL, C68:4, rose upon serum starvation and dropped upon methotrexate inhibition. Conversely, the percentage of long chain CL, C72:5, dropped upon serum starvation and rose upon methotrexate inhibition. The percentage of each CL species in the mitomycine C and vincristine treatments did not change as drastic as the species in the methotrexate group.

**Fig 7:**
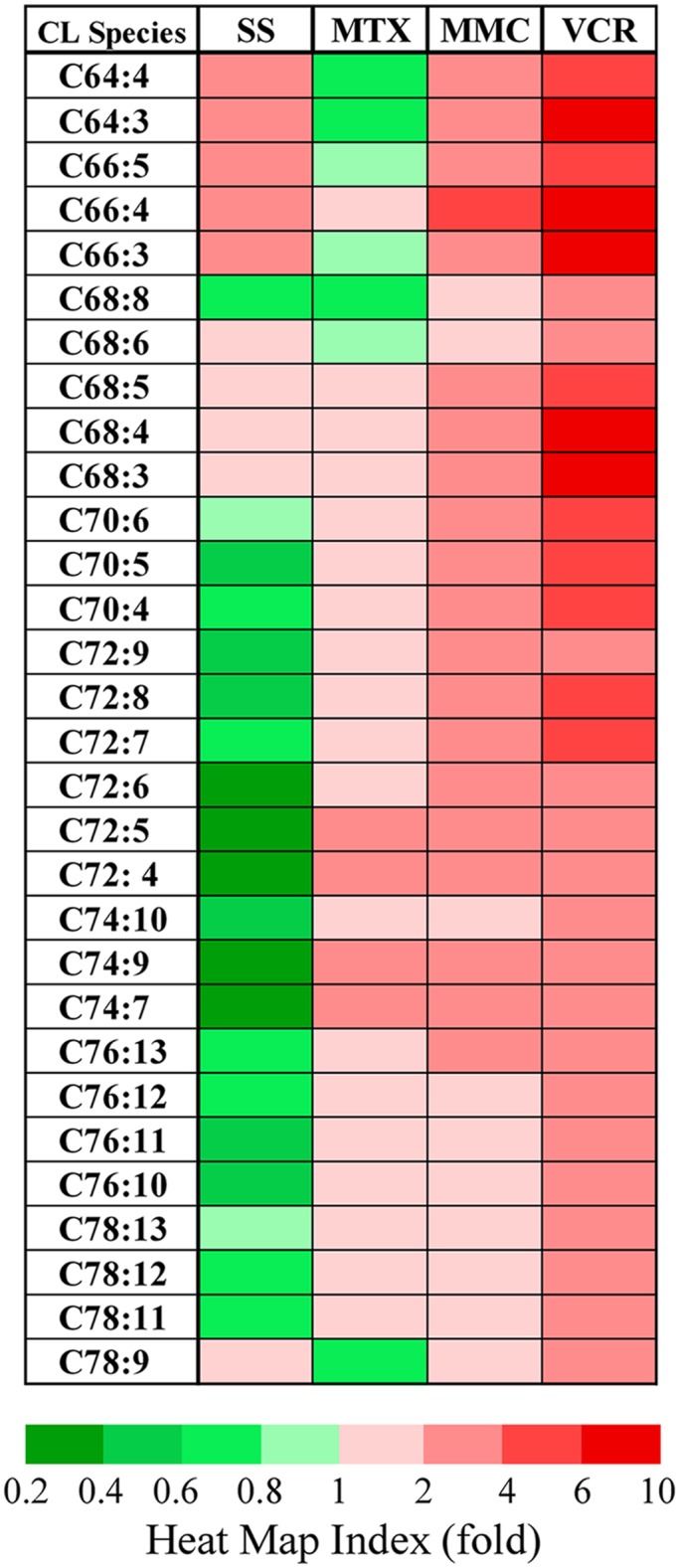
The heat map of CL quantity changes upon chemotherapy drug treatment. The quantity of each CL was semi-quantitated according to its relative ratio to the CL(14:0)_4_. The fold changes are the ratio between the treatment and the control. The ratios were displayed according to the heat map index. SS: serum starvation; MTX: methotrexate; MMC: mitomycine C; VCR: vincristine.

The total CL quantity and the percentage of each CL species in response to serum starvation and chemotherapy drugs do not represent the quantity changes of each individual species. We further examine the quantity changes of each CL species and display CL quantity changes in four types of arrests in a heat map (Fig 7). Serum starvation and MTX treatments caused the shift of cardiolipin species, which might be the results of the CL remodeling without apparent CL synthesis. However, the mitomycine C and vincristine displayed patterns of increasing CL, especially on the short chain CL, which strongly indicating a stimulation of cardiolipin synthesis and implying a shutdown of the breakdown mechanism.

## Discussion

### Discoordination of mitochondrial life cycle

Clinical chemotherapy medication not only inhibits rapid cell growth of cancer cells, but also generate stress to the normal organs where drug accumulated. Under nutritional deprivation stress, such as serum starvation, the cell cycle was arrested through AMPK activation, which resulted in the p53 phosphorylation and activation [47,48]. Without sufficient nutrition, the arrested cells shortened the chain length of cardiolipin in mitochondria and the total CL quantity decreased in the cancer cells. Because CL is an important phospholipid component in mitochondria membrane and critical for the structure and function of mitochondria, shortening the CL acyl chain is presumably related to the energy conservation purpose. Due to the coordination between the cell cycle and the mitochondrial life cycle, cell cycle arrest directly impacts the regulation of the mitochondrial life cycle [25,26]. The effects of chemotherapy drug-induced cell cycle arrest on mitochondria were evaluated by the CL quantity and species changes. Because chemotherapy drugs arrested the cell cycle with abundant nutrition, the nutrition became excessive while the cell cycle was unable to proceed. The arrested cells stopped duplicating, but continued to produce CL, leading to the significant CL quantity increases and cellular accumulation consequently. This result implies the mitochondrial life cycle discoordinated with the cell cycle, especially after the treatments of mitomycine C and vincristine. Cell cycle arrest by chemotherapy drugs did not send the signal to stop the mitochondrial life cycle and therefore, the mitochondrial life cycle escaped from the cross-talk with the cell cycle and eventually triggered the divergence between these two cycles. At this condition, mitochondria kept producing CL, resulting in excess CL to cause accumulation. It is worth to note that there is approximate 20% of CL in the total mitochondrial phospholipids. The accumulation effects could not be completely from the 2.6-fold increase of the CL percentage in one particular mitochondrion from 20% to over 50%, which is impossible to form stable membrane structure. Therefore, the observed effects ought to be at least partially, if not all contributed from the increasing quantity of mitochondria. Increase of the total CL quantity could be potentially problematic for clinical chemotherapy. Excessive CL in the humans and mice with bacterial pneumonia in lung fluid has been associated with the severity of lung injury [49]. The release of excessive CL can possibly raise the CL antibody level in patients. Chemotherapy in combination of nutritional control will be an alternative option to prevent CL accumulation.

### Fatty acyl chain changes

To summarize the remodeling changes on the acyl chains of CL in these four types of arrest mechanisms, the acyl chain indexes [50] were calculated for CL analysis (Table 1). Because the percentage of each CL has been determined, we are able to determine the average acyl chain length (ACL) in the cells. Methotrexate treatment shows 17.82 on the ACL index and serum starvation shows the lowest 17.29 on the ACL index, indicating average 0.53 carbon increases per acyl chain on CL equal to 2.12 carbons more on each CL. This result obviously shows the major shifting of the carbon chain would be between 16- and 18-carbon containing fatty acyl chain. The saturation of the fatty acyl chain can be evaluated by the double bond index DBI. The lowest double bond index DBI is 99.73 (0.99 double bond/fatty acyl chain; 3.96 double bonds/CL) in the starvation condition, indicating the decrease of the chain length also causes the decrease amount of double bonds. Interestingly, the methotrexate induced arrest shows significant decrease of the saturated fatty acyl chain

**Table 1:** Fatty acid composition of cardiolipin from HT-1080 cells after treatment of chemotherapy drugs.

	Control	Starvation	Methotrexate	Mitomycin C	Vincristine
**ACL**	17.67 ± 0.01	17.29 ± 0.02	17.82 ± 0.04	17.59 ± 0.01	17.49 ± 0.02
**DBI**	111.23 ± 0.68	99.73 ± 1.35	115.68 ± 1.48	105.21 ± 0.36	102.44 ± 0.64
**PI**	35.07 ± 0.84	27.12 ± 1.72	38.36 ± 1.73	28.24 ± 0.42	26.36 ± 0.62
**SFA**	2.84 ± 0.10	6.78 ± 0.19	1.72 ± 0.05	3.02 ± 0.11	4.09 ± 0.23
**UFA**	97.16 ± 0.10	93.22 ± 0.19	98.28 ± 0.05	96.98 ± 0.11	95.91 ± 0.23
**MUFA**	5.57 ± 0.04	5.81 ± 0.08	5.36 ± 0.08	5.93 ± 0.04	5.95 ± 0.05
**PUFA**	91.59 ± 0.10	87.40 ± 0.18	92.92 ± 0.13	91.05 ± 0.10	89.96 ± 0.22
**PUFAn-3**	0.98 ± 0.09	0.77 ± 0.06	0.92 ± 0.05	0.62 ± 0.03	0.64 ± 0.02
**PUFAn-6**	23.65 ± 0.25	17.51 ± 0.71	27.01 ± 0.78	20.33 ± 0.31	19.02 ± 0.49

Average chain length (ACL), double bond index (DBI), peroxidizability index (PI) and polyunsaturated fatty acid (PUFA) are shown. PUFAn-3: FA 18:3, 20:5, 22:5, 22:6; PUFAn-6: FA 18:2, 20:3, 20:4, 22:4, 20:2; Results in triplicate are shown as mean ± SD.

We further look into the species of changes and analyze their fatty acyl compositions. In HT-1080 cells, CL species are composed of C64, C66, C68, C70, C72, C74, C76 and C78 groups. Among them, C68, C70 and C72 are the main group. The lower carbon groups, including C64, C66 and C68 are mainly C14 and C16. The higher carbon groups, including C72, C74, C76 and C78 are mainly C16 and C18, and minor C20 and C26. The short acyl chains are mainly C16:0 and C16:1 and the long acyl chain are mainly C18:1 and C18:2. More specifically, the shifting of the fatty acyl chain is contributed most by the exchange of C16:1 and C18:1.

The fatty acyl compositions have been determined by MS/MS, the dominant species of each CL was used to calculate the rest of the indexes. The peroxidizability is following the trend of double bond index. The most intriguing results are the differences between the treatments of starvation and methotrexate. These two groups have two completely opposite and extreme indexes, reflecting the opposite effects on CL compositions in G0 and S phases.

### CL remodeling after synthesis phase

Serum starvation induced cell cycle arrest at G0 profoundly affected the compositions of CL in mitochondria in both HT1080 and MCF7 [43]. Replenishing nutrition reverted the compositions of CL after starvation. More detailed CL changes were distinguished after the cell cycle arrest at different phases, which assisted us to decipher the mechanisms of the CL remodeling. In the normal growing cells, the cells assess their health at the check points before entering next phase. From G1 to S phase, the activity of mitochondria rises to increase the efficiency of ATP production, which assists the synthesis of biological molecules for mitosis [51]. We found that methotrexate induced S phase arrest uniquely increased the long chain fatty acyl chain, which was a reverse effect from mytomycine C and vincristine induced G2/M arrest and nutritional deficiency induced G0/G1 arrest. This result indicates S phase acquires nascent CL synthesis and the remodeling occurs immediately after the completion of synthesis. S phase synthesizes most biological molecules, including lipids, proteins and DNA, which requires highly efficient mitochondria for ATP production. Long chain CL may bind to and stabilize protein complexes on the mitochondrial membrane for efficient energy production.

Major CL changes were discovered after S phase. Large amount of CL accumulation was seen on the G2/M arrest and the major changes in the CL species also occurred at this phase. CL accumulation in G2/M phase can be the result of the prolonging S phase for the unstopped CL production in mitochondria. Without completion of telophase and cytokinesis, the arrested cells cannot split the CL(s) that have been synthesized. However, the decrease of the chain length was an indication of lacking the long chain fatty acids of PG or CDP-DAG. Additionally, the short chain phospholipids incorporated into CL through the remodeling process, which conserved the usage of the carbon chain and the content of the double bonds to ensure the continuation of CL production. G2 and M phase arrests showed the same trend of total quantity and the species CL changes, indicating CL was remodeling in the S/G2 phase. After the cell cycle reached G2/M, the cells continued the process of CL synthesis and remodeling, which caused more CL accumulation. The fact that chemotherapy drug affecting the quantity and quality of mitochondria may advance CL to be a biomarker for the evaluation for chemotherapy.

## Conclusions

Chemotherapy drugs, methotrexate, mitomycine C and vincristine, arrest HT1080 human fibrosarcoma cells at S, G2 and M phases of the cell cycle respectively. Drug treatments, especially mitomycine C and vincristine, induce significant accumulation of cardiolipin (CL), indicating discoordination of mitochondrial life cycle. From the observation of the CL species changes in the four arrested phases, we also conclude the remodeling of CL is facilitated immediately after S phase.

## Supporting Information

S1 Fig Raw data of MTT assay and CL contents. (XLS)

S2 Fig Serum starvation induced CL content changes. (A) Percentage of CL species in control and after 48-hr serum starvation. Experiments are in triplicates and analyzed by t-test (*p < 0.05, **p < 0.01, ***p < 0.001). (B) The changes of CL contents after 48-hr serum starvation.
(DOC)
