# Supplementary material for: Chemotherapy Drug Induced Discoordination of Mitochondrial Life Cycle Detected by Cardiolipin Fluctuation
Source: PLoS One. 2016 Sep 14;11(9):e0162457. doi: 10.1371/journal.pone.0162457 (PMC5023183; doi:10.1371/journal.pone.0162457)
Supplement: S2 Fig — (A) Percentage of CL species in control and after 48-hr serum starvation. Experiments are in triplicates and analyzed by t-test (*p < 0.05, **p < 0.01, ***p < 0.001). (B) The changes of CL contents after 48-hr serum starvation. (DOC) [file pone.0162457.s002.doc]

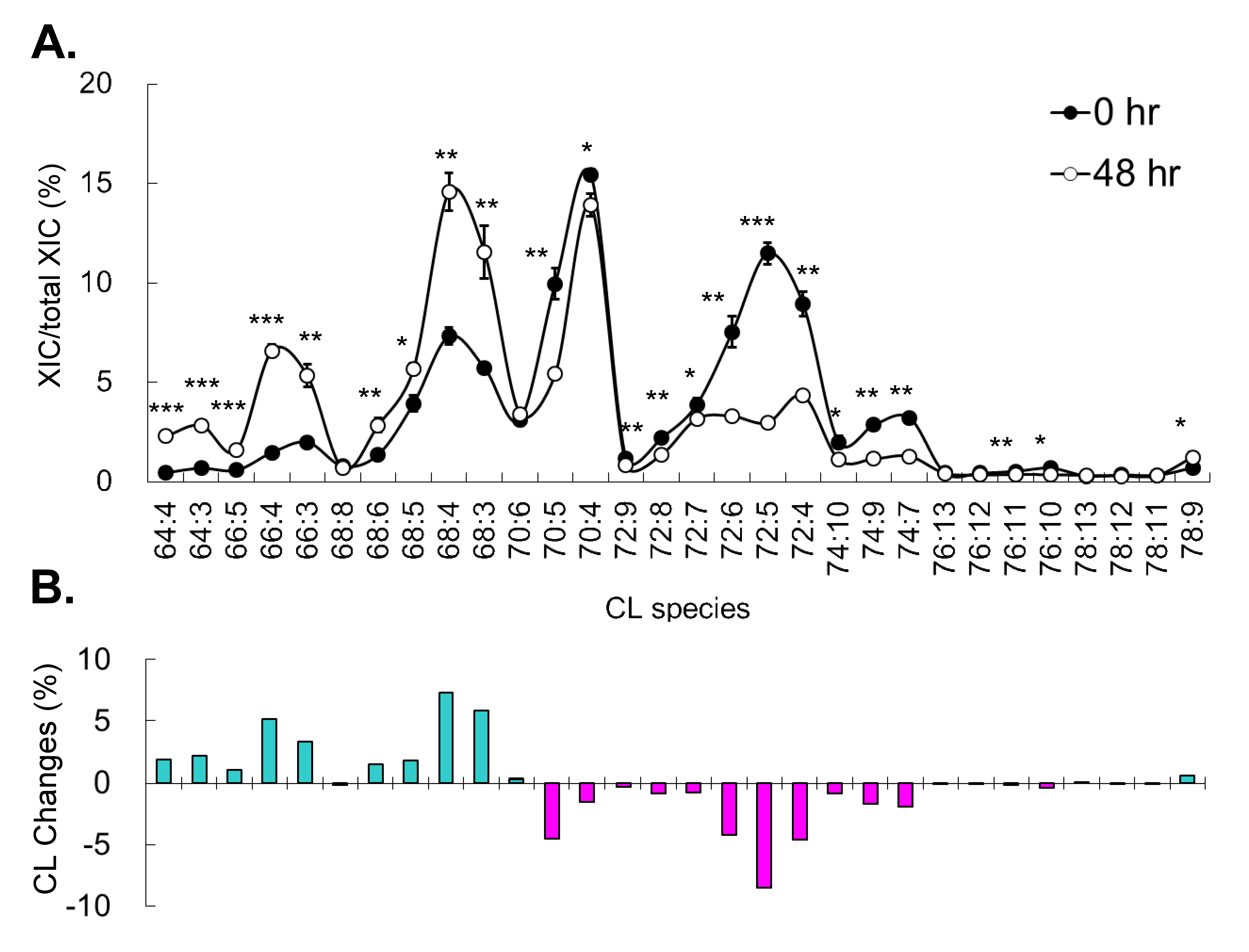


**S2 Fig. Serum starvation induced CL content changes.** (A) Percentage of CL species in control and after 48-hr serum starvation. Experiments are in triplicates and analyzed by t-test (**p* < 0.05, ***p* < 0.01, ****p* < 0.001). (B) The changes of CL contents after 48-hr serum starvation.
